# Clinical Trial of Efficacy Evaluation of Omega-3 with Risperidone on Seizures Frequency in Children with Refractory Epilepsy and Attention-Deficit/Hyperactivity Disorder

**Published:** 2018

**Authors:** Razieh FALLAH, Shiva EILIAEI, Farzad FERDOSIAN

**Affiliations:** 1 Department of Pediatrics, Growth Disorders of Children Research Center, Shahid Sadoughi University of Medical Sciences, Yazd, Iran; 2 General Physician, Shahid Sadoughi University of Medical Sciences, Yazd, Iran

**Keywords:** Epilepsy, Refractory epilepsy, Omega-3, ADHD

## Abstract

**Objectives:**

We aimed to answer the question whether or not previous antiepileptic drugs with combination of omega-3 and risperidone are more efficient than previous antiepileptic drugs with risperidone alone in decreasing of seizures monthly frequency of children with refractory epilepsy and attention-deficit/hyperactivity disorder (ADHD).

**Material & Methods:**

In a randomized clinical trial (IRCT201604212639N18), participants referred to Pediatric Neurology Clinic of Shahid Sadoughi Hospital, Yazd, Iran from Jun 2015 were distributed randomly into two groups. In group I, one capsule of omega-3 daily and 0.5 mg of risperidone was divided into two doses with previous antiepileptic drugs and in group II, 0.5 mg of risperidone was divided into two doses with previous antiepileptic drugs were given. The drugs use was continued for three months and the children were followed up monthly for three consecutive months. Primary outcomes included seizure monthly frequency and good response (more than 50% of reduction in seizures monthly frequency). Secondary outcome was clinical side effects.

**Results:**

Overall, 23 girls and 33 boys with mean age of 9.24+0.15 yr (29 children in omega-3 group and 27 children in control group) were evaluated. Omega-3 therapy was effective in decreasing of seizures monthly frequency (10.41±3.92 times vs. 17.01±4.98, *P*=0.03). Good response was seen in three children (11.1%) in control (95% confidence interval: 8%-22.8%) and in 9 children (31%) in omega-3 (95% CI: 47.83%-14.17%) group, which showed that omega-3 was more effective in seizure control. (*P*=0.001). Frequency of side effects was not different in the two groups (14.8 % in control vs. 20.7% in omega-3 groups, *P*=0.5).

**Conclusion:**

Omega-3 might be used as an effective and safe drug in seizures control of children with refractory epilepsy and ADHD.

## Introduction

Epilepsy defined as at least two unprovoked seizures occurring at least 24 h apart, has a cumulative lifetime incidence of 3% and annual prevalence of 0.5%-1% (1). Intractable or refractory epilepsy defined as recurrence of at least one seizure in a week in spite of taking two or three appropriate antiepileptic drugs with sufficient dosage and includes approximately a third of newly treated epileptic (2-4).

On the other hand, attention-deficit/hyperactivity disorder (ADHD) characterized by inattention, including increased distractibility and difficulty sustaining attention, poor impulse control and decreased self-inhibitory capacity, and motor overactivity and restlessness, is the most common neurobehavioral disorder of childhood (5) continued to adult and an adult ADHD types include inattentive and emotional dysregulation (6). Worldwide-pooled prevalence of ADHD in a meta-analysis study was 3.4% (7). Epileptic children have more ADHD-related symptoms and association between epilepsy and ADHD (8-11).

The three most important polyunsaturated fatty acid of omega-3 including alpha-linolenic, eicosapentaenoic and docosahexaenoic acids are necessary for the correct function of the organism and take part in many brain physiological processes. The body cannot synthesize these omega-3 fatty acids in enough amounts, and therefore they must be added to the diet and omega-3 polyunsaturated fatty acids may inhibit neuronal excitability and may have anticonvulsant effects and a potential treatment use of medically resistant epilepsy (12). 

Eicosapentaenoic acid alone (13), eicosapentaenoic acid plus docosahexaenoic acid (14) and omega-3 polyunsaturated fatty acids (15) were effective in reduction of severity or frequency of epileptic seizures. On the other hand, ratios of both blood omega-6 to omega-3 and arachidonic acid to eicosapentaenoic acid have been elevated in ADHD children (16). A clinical trial study showed that dietary supplementation with omega-3 fatty acids reduced ADHD symptoms (17). 

Effect of omega-3 fatty acids on seizures control and omega-3 efficacy in reduction of ADHD symptoms have been assessed in other studies, but, we did not find any research which evaluated effectiveness of these fatty acids in children with combination of refractory epilepsy and ADHD. Therefore this clinical trial was conducted to answer the question whether or not previous antiepileptic drugs with combination of omega-3 and risperidone is more efficient than previous antiepileptic drugs with risperidone alone in decrease of seizures monthly frequency of children with refractory epilepsy and ADHD.

## Materials & Methods

In a randomized single-blinded parallel group, clinical trial, all consecutive 7-11 yr old children with combination of refractory epilepsy and ADHD referred to Pediatric Neurology Clinic of Shahid Sadoughi Hospital, Yazd, Iran from Jun 2015, were enrolled. Sample size was determined by help of statistical consultant based on Z formula and a confidence interval of 95% with 80% power type one error of 5%, and an effect size (difference in frequency of good response between the two groups) of 30% based on result of our pilot study, was assessed in 30 children in each group.

Inclusion criteria for eligible participants we as follows: Children aged 7-11 yr, had refractory epilepsy based on definition of the International League against Epilepsy (1), had ADHD based on DSM-IV criteria, had at least score of 20 in ADHD diagnostic rating scale via parent interview (5) and were able to walk. Exclusion criteria consisted of receiving all kinds of supplement within the past two months, other psychiatric disorders, status epilepticus during research period, allergy to omega-3 capsule or risperidone, change in antiepileptic drugs regimen, use of phenobarbital or topiramate, irregular drugs usage and discontinuation of omega-3 capsule or risperidone usage for more than one week.

The trial used computer-generated equal simple randomization by random numbers and allocation ratio was 1:1 for the two groups. Randomization and blinding were done by an investigator with no clinical involvement in the trial. Data collectors, outcome assessors, and data analysts were all kept blinded to the allocation. Concealment was done by placing the group number for each serially participating child in a numbered and sealed opaque envelope opened by the pediatric neurologist of research immediately before study enrollment. The drug was delivered by the mothers of the patients and primary and secondary outcomes were assessed by the intern of research not informed of the drug group assignment. The children were randomly distributed into two groups. In group, I, one capsule of omega-3 daily and 0.5 mg of risperidone divided into two doses with previous antiepileptic drugs and in group II, 0.5 mg of risperidone divided into two doses with previous antiepileptic drugs, were given. The drugs use was continued for three months and the children were followed up monthly for three consecutive months. 

The capsule of omega-3 that used in this research was fish oil (Omega-3) from 21st Century Co, the USA that each capsule contains 1000 mg of omega 3 fish oil, 180 mg of eicosapentaenoic acid and 120 mg docosahexaenoic acids. Moreover, risperidone tablet of 1 mg was from Abidi Co, Iran.

Primary outcomes included seizure monthly frequency and good response (more than 50% of reduction in seizures monthly frequency) compared before and after three months of treatment. Secondary outcome was clinical side effects.

The data were analyzed using SPSS version 17 (Chicago, Illinois, USA) statistical software. Recorded data were assessed for normal distribution using the Kolmogorov-Smirnov test and Chi-square test was used for data analysis of categorical variables and continuous and mean variables were compared using independent *t*-test between the two groups. Differences were considered significant at *P*-values of less than 0.05.

Informed consent was taken from the parents of the children before enrolling and this study has been approved by the Ethics Committee of Shahid Sadoughi University of Medical Sciences, Yazd, Iran. This research is registered in Iranian Clinical Trials (www.irct.ir) under registration number: IRCT201604212639N18.

## Results

**Fig 1:**
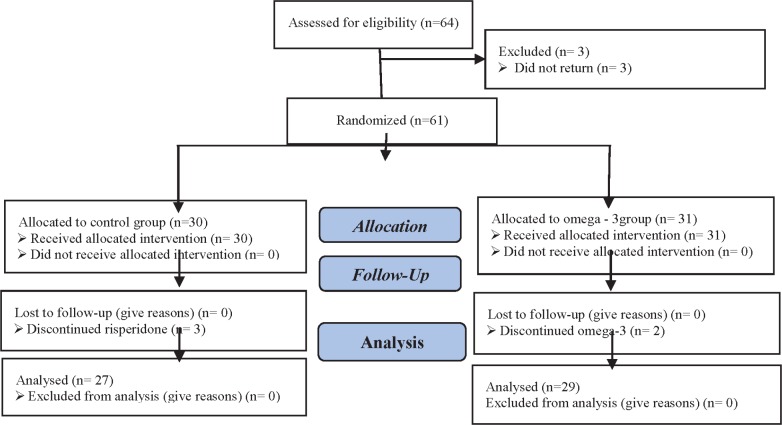
CONSORT flow diagram

Sixty-four children were enrolled, but three children did not return and five children stopped taking medications after 3-4 wk. Therefore, they were excluded and finally, 56 children including 23 girls and 33 boys with mean age of 9.24±0.15 (29 children in group of omega-3 and 27 children in control group) were evaluated in the two groups (Figure 1). By Kolmogorov-Smirnov test, the data had normal distribution. 

Comparison of some characteristics of the children in the two groups is shown in Table 1 which indicates that no statistically significant differences were seen from viewpoints of sex distribution, seizure type, epilepsy classification and developmental status.

Table 2 shows comparisons of the mean of age, weight, and seizure monthly frequency in the two groups and indicates that no statistically significant differences were seen from these viewpoints.

Comparison of frequency of good response and monthly seizure frequency at the end of research period in both groups is shown in Table 3 which shows that omega-3 therapy was effective in decreasing of monthly frequency of seizures. More than 50% of reduction in monthly seizure frequency (good response) occurred in 3 children (11.1%) in control (95% confidence interval: 8%-22.8%) and in 9 children (31%) in omega-3 (95% CI: 47.83%-14.17%) group, respectively, combination of omega-3 and risperidone with previous antiepileptic drugs was more effective in controlling of seizures. (*P*-value=0.001)

Side effects included sleepiness in two children, anorexia in one child and constipation in one child were seen in 14.8% of control group and sleepiness in two children, diarrhea in two children and nausea and vomiting in two children (20.7%) were seen in omega-3 group and frequency of adverse events was not different in the two groups (*P*=0.5).

## Discussion

Based on our results, daily usage of 1000 mg of omega-3 fish oil, 180 mg of eicosapentaenoic acid and 120 mg docosahexaenoic acids for 12 wk with combination of previous antiepileptic drugs and risperidone was more efficient than previous antiepileptic drugs and risperidone alone in reducing of seizures monthly frequency of children with refractory epilepsy plus ADHD. 

We did not find any research that evaluated the efficacy of omega-3 in children with refractory epilepsy and ADHD.

**Table 1:** Comparison of some characteristics of children in the two groups

Groups		Control	Omega- 3	*P*-value
Data				
Sex	Girl	10	13	0.9
	Boy	17	16	
Seizure type	Generalized	13	12	0.7
	Partial	4	6	
	Mixed	10	11	
Epilepsy classification	Symptomatic	14	17	0.8
	Cryptogenic	8	9	
	Idiopathic	5	3	
Developmental status	Delay	19	19	0.9
	Normal	8	10	

**Table 2:** Comparison of mean of age, weight and seizure monthly frequency in the two groups

Groups	Control	Omega- 3	*P*-value
Data			
Age in years (mean ± SD)	10.57 ± 2.44	10.11 ± 2.13	0.4
Weight in kilogram (mean ± SD)	8.59 ± 1.56	8.34 ± 2.45	0.6
Monthly seizure frequency (mean ±SD)	16.7 ± 6.68	15.8 ± 8.49	0.09

**Table 3:** Comparison of frequency of good response and monthly seizure frequency in both groups

Groups		Control	Omega- 3	*P*-value
Data				
Good response (> 50% of reduction in monthly seizure frequency)	Yes	3	9	0.001
	No	24	20	
Monthly seizure frequency at the end of research (mean±SD)		17.01±4.98	10.41±3.92	0.03

In a case-control study in Alexandria, Egypt, the efficacy of omega-3 supplements as fish oil in decrease of frequency and severity of epileptic seizures of 70 children with refractory epilepsy were evaluated. Omega-3 caused significant reduction in seizure frequency and fish oil could elevate the seizure threshold in medically resistant epileptic children, but there was not any statistically significant difference in seizures severity improvement between cases and control (18). 

In California, USA, the efficacy of high dose and low dose of fish oil versus placebo (corn oil, linoleic acid) in 24 patients with refractory epilepsy was compared and low-dose fish oil (3 capsules/d, 1080 mg eicosapentaenoic acid plus docosahexaenoic acid) was more effective than placebo in reduction of seizures. High-dose fish oil did no cause difference than placebo in reducing seizures (19). In London, UK, daily usage of 1000 mg of eicosapentaenoic acid for 3 months caused 12%-56% reduction in seizure frequency in six from ten patients and in one other person had markedly reduced seizure severity (13).

Efficacy of 12 wk treatment of daily one gram of eicosapentaenoic acid and 0.7 gr docosahexaenoic acid on 57 epileptic patients were evaluated by randomized, placebo-controlled clinical trial. “Seizure frequency was reduced over the first 6 wk of treatment in the supplement group, but this effect was not sustained” (14).

Significant reduction in both frequency and severity of seizures occurred in all five epileptic patients with central nervous system diseases treated with 5 gr of omega-3 polyunsaturated fatty acids at every breakfast for 6 months (15).

A 7-year-old boy with Lennox-Gastaut syndrome and refractory epilepsy was reported successfully treated with a polyunsaturated fatty acid -enriched modified Atkins diet without any life-threatening side effects (20). 

However, in USA, eicosapentaenoic acid plus docosahexaenoic acid, 2.2 mg/day in a 3:2 ratio for 12 wk was not superior to placebo in twelve adults with refractory focal or generalized epilepsy (21). A possible explanation for this discrepancy may be related to differences in the age of the patients, dosage of omega-3, ratio of eicosapentaenoic acid to docosahexaenoic acid ratios and treatment duration.

Approximately 60% of the dry weight of brain includes lipids and about 30% of brain lipid consists of polyunsaturated fatty acids (omega-3, omega-6...) and omega-3 can regulate neuronal function via stabilizing neuronal membranes by inhibiting the voltage-dependent sodium and calcium currents, modulating of membrane biophysical properties, regulation of neurotransmitter release, signaling of neurotransmitter and synthesis of biologically active oxygenated derivatives and possible antiseizure effects of omega-3 may be due to increasing in seizure thresholds and lowering of inflammatory cytokines in epileptic patients (22) and by these mechanisms, omega-3 fatty acids can decrease seizure-associated cardiac arrhythmias and sudden cardiac deaths in epileptic patients (23).

In this study, omega-3 was safe and no life-threatening clinical side effect was seen in children. Safety of omega-3 even at high doses has also been reported in other studies (18, 19-21).

**In conclusion,** our results are promising and omega-3 can be considered as an effective and without life-threatening side effects drug in control of seizures in children with refractory epilepsy and ADHD. It is worth to do other clinical trials with larger sample sizes, different omega-3 fatty acid preparations, different doses and longer treatment duration.
